# Orthopedic management of the extremities in patients with Morquio A syndrome

**DOI:** 10.1007/s11832-014-0601-4

**Published:** 2014-07-08

**Authors:** Klane K. White, Andrea Jester, C. Edward Bache, Paul R. Harmatz, Renée Shediac, Mihir M. Thacker, William G. Mackenzie

**Affiliations:** 1 Department of Orthopaedic Surgery and Sports Medicine, Seattle Children’s Hospital, University of Washington, Seattle, WA USA; 2 Hand and Upper Limb Service, Dept. of Plastic Surgery, Birmingham Children’s Hospital, Birmingham, UK; 3 Department of Orthopaedics, Birmingham Children’s Hospital, Birmingham, UK; 4 UCSF Benioff Children’s Hospital Oakland, Oakland, CA USA; 5 BioMarin Pharmaceutical Inc, Novato, CA USA; 6 Department of Orthopaedic Surgery, Nemours/A.I. duPont Hospital for Children, Wilmington, DE USA

**Keywords:** Morquio A, Mucopolysaccharidosis IVA, MPS IVA, Lower extremity, Upper extremity

## Abstract

**Background:**

Musculoskeletal involvement in Morquio A syndrome (mucopolysaccharidosis IVA; MPS IVA) contributes significantly to morbidity and mortality. While the spinal manifestations of the disorder have received considerable attention in the literature, there have been few reported studies to date to guide the management of the orthopedic problems associated with the lower and upper extremities.

**Purpose:**

The objective was to develop recommendations for the management of the extremities in patients with Morquio A syndrome.

**Methods:**

A group of specialists in orthopedics, pediatrics and genetics with experience in the management of Morquio A patients convened to review and discuss current clinical practices and to develop preliminary recommendations. Evidence from the literature was retrieved. Recommendations were further refined until consensus was reached.

**Results and conclusions:**

This present article provides a detailed review and discussion of the lower and upper extremity deformities in Morquio A syndrome and presents recommendations for the assessment and treatment of these complications. Key issues, including the importance of early diagnosis and the implications of medical therapy, are also addressed. The recommendations herein represent an attempt to develop a uniform and practical approach to managing patients with Morquio A syndrome and improving their outcomes.

## Introduction

Morquio A syndrome, or mucopolysaccharidosis IVA (MPS IVA), is an autosomal recessive lysosomal storage disorder caused by defective activity of *N*-acetyl-galactosamine-6-sulfatase (GALNS), an enzyme that catabolizes the glycosaminoglycans keratan sulfate and chondroitin-6-sulfate. Symptoms typically become apparent during the first few months or years of life, as intracellular glycosaminoglycan accumulation progressively affects skeletal structure, connective tissues and organs. Over 275 mutations in the *GALNS* gene have been identified to date, resulting in wide phenotypic variability [1–4]. The condition is rare, with estimates of incidence ranging from one in 76,000 live births in Northern Ireland [5] to one in 640,000 live births in Western Australia [6].

Musculoskeletal manifestations, including short stature, spine abnormalities, hip dysplasia, genu valgum, joint laxity and abnormal gait, are usually among the first symptoms to develop [7–9]. While the natural history of Morquio A syndrome has not been well defined, musculoskeletal involvement is progressive and patients typically require multiple orthopedic interventions to prevent deformity and to improve function [7–10]. Spinal involvement is common and can lead to irreversible neurological damage and premature death [11]. Progressive lower extremity involvement and increasing muscle weakness compromise mobility; consequently, many patients require the use of walking aids or become wheelchair-bound by adolescence [7–10]. The hands are frequently involved and gradual declines in grip strength, wrist stability and fine motor skills lead to significant functional compromise [12]. Non-skeletal manifestations, including impaired respiratory function and cardiac valve disease, also contribute significantly to morbidity and mortality [9, 13]. The management of Morquio A syndrome has typically been supportive and symptom-based, but enzyme replacement therapy (ERT) has recently become available [14], underscoring the importance of early and accurate diagnosis. However, recognizing and correctly diagnosing affected individuals, particularly those with non-classic phenotypes, can be challenging [15, 16].

Although orthopedic care is a key component of Morquio A syndrome management, guidelines for treatment and surgical decision-making remain scarce. Recommendations for the diagnosis and management of spinal involvement in Morquio A syndrome were recently published [11]. However, limited evidence is currently available in the literature to guide the management of the extremities in these patients. With the aim of improving patient outcomes, this article provides a focused and detailed review and discussion of the orthopedic complications associated with lower and upper extremity involvement in Morquio A syndrome, and presents recommendations for their management based on current evidence and the experience of the authors. Additional key issues in the management of this disorder, including the challenges of diagnosis and the implications of medical therapy, are also discussed.

## Methods

The authors met in August 2013 to initiate the formulation of recommendations for the orthopedic management of the extremities in Morquio A patients. This meeting was sponsored by BioMarin Pharmaceutical Inc. Current clinical practices were reviewed and discussed; the institutions represented by the authors are well-established referral centers for MPS and collectively manage, have evaluated, or completed surgeries for over 100 Morquio A patients. A preliminary outline of recommendations was drafted during the meeting. A review of the literature was conducted to identify and gather relevant evidence. Due to the limited available literature, all articles related to orthopedic management of the extremities in Morquio A syndrome, including single case reports, were reviewed. Recommendations were refined by the authors and finalized during the development of this manuscript.

## Pathophysiology

Historically viewed as a skeletal disorder, Morquio A syndrome tends to manifest predominantly as bone dysplasia and short stature. While the mechanisms underlying growth plate pathophysiology are currently not well understood, histological studies show that articular cartilage is altered; specifically, keratan sulfate accumulation in chondrocytes, poorly organized tissue structure, increased type I collagen and reduced type II collagen, and thick irregularly shaped collagen fibrils have been reported [17–20]. Glycosaminoglycans influence a variety of biological pathways, and there is evidence to suggest that keratan sulfate accumulation modifies the expression of chondrogenic genes [21]. Insight from other MPS animal models suggests that glycosaminoglycan-mediated inflammation may also play a significant role in growth plate dysfunction [22, 23].

## Diagnosis

With the recent availability of ERT for Morquio A syndrome, early and accurate diagnosis becomes critical for improved patient outcomes. Because skeletal abnormalities are common initial presenting symptoms and typically bring a patient to medical attention, orthopedic surgeons are uniquely positioned to ensure a timely and accurate diagnosis of this disorder. Classic clinical signs and symptoms include marked short stature, genu valgum, kyphoscoliosis, pectus carinatum, abnormal gait and joint laxity. These findings are frequently not evident until the third or fourth year of life. Typically, the earliest musculoskeletal manifestation is the characteristic thoracolumbar gibbus, which is often evident in the first 6 months. Familiarity with the radiographic manifestations of dysostosis multiplex aids the recognition of a possible MPS disorder [24]. Classic radiographic findings include abnormally shaped vertebral bodies with anterior beaking, posterior scalloping, platyspondyly, dens hypoplasia, short ulna, steep radius, short and broad metacarpals with proximal rounding, delayed carpal bone maturation, rounded iliac wings, inferiorly tapered ilia, and hypoplastic epiphyses [24–26]. In practice, however, there is wide variability in presentation, leading to the potential for a delayed or missed diagnosis. In patients with non-classic phenotypes, the radiographic manifestations associated with dysostosis multiplex may be subtle or absent, and epiphyseal and/or vertebral changes may resemble those of other skeletal disorders/dysplasias [25]. Recent case series that highlight the challenges of diagnosing Morquio A syndrome suggest that MPS should be considered in suspected cases of multiple epiphyseal dysplasia, spondyloepiphyseal dysplasia and bilateral Perthes-like disease [15, 16]. Consideration of the overall clinical picture is also important, as non-skeletal manifestations such as corneal clouding, umbilical hernias and cardiac valve disease can yield vital clues for achieving a correct diagnosis. Once clinical suspicion of an MPS disorder has been established, the patient should be referred to a geneticist for biochemical testing. Diagnostic enzyme testing is required for a definitive diagnosis of Morquio A syndrome, since urinary glycosaminoglycan analysis alone can be misleading [15, 16, 26]. Significantly reduced or absent GALNS enzyme activity in isolated leukocytes or cultured skin fibroblasts is diagnostic of Morquio A syndrome; however, additional enzyme analyses to rule out Morquio B syndrome and multiple sulfatase deficiency are highly recommended for definitive diagnosis [26]. Molecular analysis can also be useful in confirming a diagnosis of Morquio A syndrome if two known causative mutations can be identified [26]. A diagnostic testing algorithm has recently been published to streamline this complex process [26].

## Growth

Patients with “classic” Morquio A syndrome generally have very short stature. Although growth is typically normal during infancy, growth velocity usually starts to decline after the age of 1 year [27]. By the age of 3 years, growth delay is evident. As a result, standard atlases cannot be used to stage growth in these patients. Patients with “non-classic” Morquio A syndrome often present with normal stature that fails to stay within normal limits during the pre-adolescent growth spurt [15]. Consequently, determination of bone age and growth potential is difficult and must be considered when formulating treatment plans. Growth charts for Morquio A syndrome have been developed and can offer some guidance in this regard [27]. Familiarity with the phenotypic variability in Morquio A syndrome is required for interpretation of these growth charts. While patients with a “classic” phenotype follow a generally predictable growth pattern, others may demonstrate wide variability.

## Lower extremity involvement

### Hip dysplasia and subluxation

**Fig. 1:**
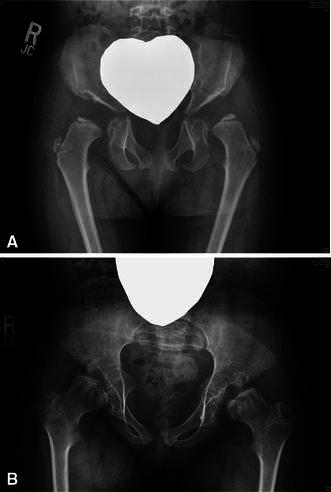
Progression of hip deformities in Morquio A syndrome. **a** 3-year-old female with dysplastic proximal femoral epiphyses and dysplastic acetabulum. Subluxation, as evident by the break in Shenton’s line, is present at this early age. **b** A similar deformity is found in an untreated 8-year-old with worsening epiphyseal flattening and incongruent acetabulum

Morquio A patients typically present with abnormal ossification of the proximal femoral epiphysis, mildly subluxated hips with a shallow and small acetabulum, and femoral neck valgus (Fig. 1). Initial development of the hip is broadly normal, but in severe cases, changes may develop as early as the second year of life. Progressive collapse and flattening of the ossific nucleus of the proximal femoral epiphyses are observed over time. Anterior superior deficiency of the acetabulum exists with a general appearance of a shallow, dysplastic acetabulum. Femoral head coverage progressively decreases with age, resulting in progressive subluxation of the hips. The native acetabulum becomes progressively shallower and is eventually lost with development of a pseudo-acetabulum. Knee deformity (genu valgum) may accelerate these hip deformities. Left untreated, hip subluxation predisposes patients to degenerative arthritis.

### Genu valgum

**Fig. 2:**
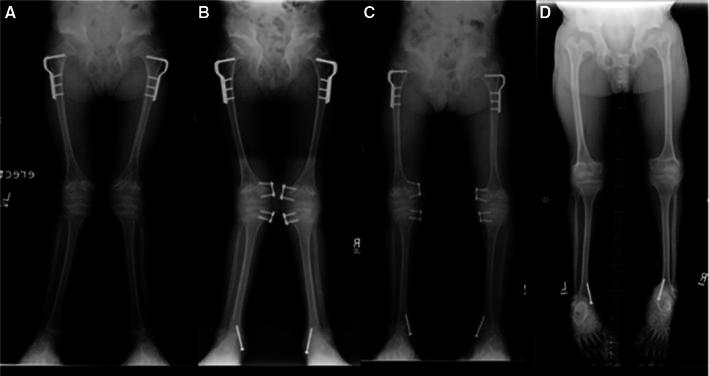
Genu valgum is common in Morquio A syndrome and often requires surgical correction. Guided growth using the tension band plate technique can be effective for genu valgum correction in younger patients with adequate growth potential. A patient who underwent surgical intervention for genu valgum **a** at age 9 years, **b** at age 10 years, **c** at age 12 years (slight over-correction, tension band plates removed) and **d** at age 16 years (skeletally mature)

The knee is the most commonly affected lower extremity joint in Morquio A syndrome [7–9]. Genu valgum typically progresses once ambulation commences, and usually becomes severe enough to warrant surgery (Fig. 2). Laxity of the collateral ligaments aggravates the deformity. The proximal tibia appears to contribute more deformity than the distal femur. There may also be procurvatum of the distal femur with recurvatum of the proximal tibia. Arthrograms or magnetic resonance imaging (MRI) of the knees in these patients show the proximal lateral portion of the tibia to be unossified and the fibula to be short, and can offer clarity as to the bony or cartilaginous nature of the deformity [8]. Progressive collapse and fragmentation of the ossific portion of the femoral and tibial epiphysis resembles that of the hip.

### Ankle valgus and foot deformities

The ankles of Morquio A patients are typically in valgus with wedging of the distal tibial epiphysis and shortening of the fibula. The severity of the ankle valgus is variable and may progressively worsen over time. There may be hindfoot valgus, some degree of equinus in the hindfoot, and adductus in the forefoot. There is some degree of supination with first metatarsal head prominence dorsally. On lateral views, tarsal bones are irregular and the talus appears plantarflexed.

### Abnormal gait

**Fig. 3:**
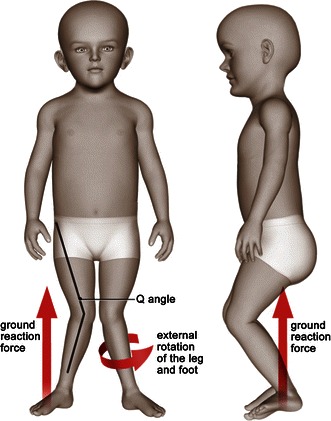
Malalignment of the knee joint alters the biomechanics of gait in patients with Morquio A syndrome. Knee valgus causes the ground reaction force to be shifted laterally, which forces the knee into further valgus. The degree of genu valgum may be estimated by the quadriceps angle, or *Q* angle (the angle formed by a line drawn from the anterior superior iliac spine through the center of the patella and a line drawn from the center of the patella to the center of the tibial tubercle). Multiple anatomical factors contribute to the magnitude of the *Q* angle, including hip rotation, tibial torsion and foot position

Reduced speed, cadence and stride length are primary gait abnormalities associated with Morquio A syndrome [28]. Lower extremity malalignment, muscle weakness and joint laxity contribute to the pathological gait pattern. Knee valgus causes the ground reaction force to be shifted laterally, which forces the knee into further valgus (Fig. 3). The extensor mechanism may subluxate laterally, resulting in reduced extensor mechanism moment arm. In severe cases, the ground reaction force moves behind the center of the rotation, necessitating quadriceps activity throughout stance phase, and may cause a flexion contracture of the knee. As individuals develop progressive genu valgum and subluxation of the extensor mechanism, there is a tendency for the tibia to follow into external rotation. This results in further reduction in power generation at the ankle. Eventually, the mechanics become so abnormal and power generation so poor that individuals cease ambulating.

## Upper extremity involvement

### Bony anomalies

**Fig. 4:**
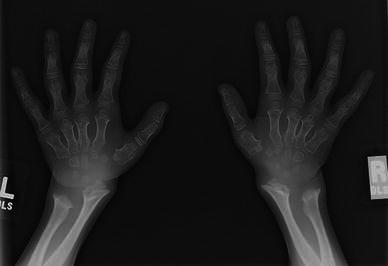
Hand and wrist changes in Morquio A syndrome. Carpal ossification delay, short and broad metacarpals, metacarpal rounding, and a short ulna are evident in the radiograph of an 8-1/2-year old female.

Characteristic radiographic findings in the hands and distal forearms of individuals with Morquio A syndrome (Fig. 4) include a short ulna, ulnar deviation of the radial epiphysis, and delayed maturation of the carpal bones; the scaphoid may not be radiographically present [25, 29]. Metacarpals may be short and the proximal ends of the second through fifth metacarpals are typically rounded (or pointed) [25, 29].

### Wrist hypermobility and reduced strength

**Fig. 5:**
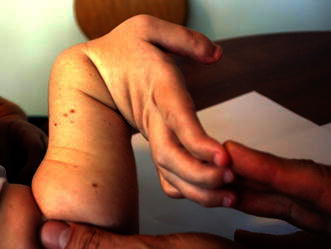
Hypermobility and ulnar deviation of the wrist joint are characteristic features of Morquio A syndrome

Clinical examination of the hands of Morquio A patients usually reveals a hypermobility of the wrist joint with a characteristic ulnar deviation (Fig. 5) that affects the active and passive joint range of motion (ROM). Patients typically show significant differences in active and passive ROM at the wrist joint, reflecting the loss of stability at this joint [12]. It is unclear if the hypermobility is due to abnormalities of the bone, ligaments, and/or muscles. A likely result of this hypermobility is the reduced strength of the wrist, metacarpophalangeal, and proximal interphalangeal joints. Patients typically show a pronounced decrease in grip and pinch strength and have difficulties performing day-to-day tasks requiring strength such as pouring from a bottle, carrying objects, and using a fork and knife [12]. In contrast, patients usually do not show significant impairment in performing tasks that involve pressing buttons or typing, such as using computers or mobile devices, over short periods [12].

## Diagnostic and monitoring tools

### Lower extremity

#### Physical examination

Physical examination of the lower extremities should include evaluation of overall alignment, joint ROM, and gait pattern. Alignment of the knees, ankles and feet typically reveals valgus deformities. Hip ROM should include prone internal and external rotation, as well as a Thomas test for hip flexion contractures. Knee ROM may reveal flexion contractures. Assessment of the intermalleolar distance (both standing and supine) is a good method of clinically assessing genu valgum. Ligamentous laxity, particularly of the medial collateral ligament and cruciates, is common. Gait pattern, as discussed previously, results from progressive knee extensor dysfunction. Myelopathic gait (broad based gait with decreased cadence) may develop with spinal cord compression. Further detailed neurological examination including evaluation of strength, sensation (including vibratory discrimination) and reflexes should also be performed every 6 months for the first 5 years of life, and then yearly thereafter.

#### Imaging

Radiography is the standard imaging modality for evaluating lower limb alignment. Pelvis and standing full-length anteroposterior radiographs should be obtained at the time of diagnosis. Radiographic analyses should include mechanical axis zone measurements as defined by Stevens [30], knee and ankle measurements, including the tibiofemoral angle (TFA), mechanical lateral distal femoral angle (LDFA) and medial proximal tibial angle (MPTA), as described by Paley et al. [31], and pelvis measurements including neck shaft angle, femoral head coverage, and acetabular index. Follow-up radiographic studies should be performed on patients who show signs of progressive hip dysplasia, genu valgum or gait abnormalities on physical examination.

Although radiographs are the mainstay for assessing and monitoring lower extremity bone involvement, they are limited in their utility for preoperative planning. Two-dimensional computed tomography (CT), which provides detailed information on bone structure and allows precise determination of bony dimensions, is recommended for pre-operative decision-making and planning for acetabular reconstructive procedures [32]. CT studies are also helpful for evaluating static torsional alignment. Progressive fragmentation and collapse of the osseous structures can lead to false assumptions regarding the anatomy around joints. Arthrography or MRI can give a more accurate picture of the non-ossified structures and the articular surfaces. The cartilaginous part of the epiphysis is often not as deformed as would be expected by the plain radiographs.

#### Gait analysis

Instrumented gait analysis can be a useful tool for pre-operative planning and for objectively assessing surgical outcomes. It enables identification of the factors that contribute to impaired gait, including rotational malalignment that cannot be determined from weight-bearing standing radiographs. Torsional and angulatory malalignments may be magnified during gait, and as such, the dynamic nature of gait analysis provides additional information to static examinations such as MRI or CT. However, the acquisition and analysis of the kinematic and kinetic data are time-consuming and currently limit the utility of gait analysis as a routine functional assessment and monitoring tool. As gait analysis is not available at many institutions, referral of a patient to a specialty center should be considered when gait studies are deemed valuable.

The group consensus is that it is advisable to correct deformity (particularly around the knee) early. In young children (<5 years of age), gait deviations will be minimal and gait analysis is probably not necessary. Older children may either present with severe gait disturbance or may have developed recurrent deformity. In such cases, gait analysis serves several purposes. Many children can have deformity at the hip, knee and ankle levels. In addition, deformity occurs in the sagittal, coronal and transverse planes. Interpreting such complex deformity by simple observation is difficult. Gait analysis not only allows kinematic data to be examined, but also kinetic data. Appreciation of power generation and moment arms at the joints is important, since injudicious surgical intervention may render more severely involved children immobile. The aim of surgery is to maintain the ground reaction force as close to the center of rotation of the joint as possible, and this may involve correction of deformity at several levels and in several planes. Pre-operative assessment of this is only possible with instrumented gait analysis. Equally important is the objective measurement of post-operative results.

### Upper extremity

#### ROM and strength

Regular assessments of ROM and strength provide insight into the degree and progression of functional impairment of the hands. Both active and passive ROM of the wrists and digits should be determined using a goniometer. Grip strength should be assessed using a dynamometer, and pinch strength should be measured using a pinch meter. ROM of the elbows and shoulders may also be examined if deemed useful.

#### Functional assessments

Assessments of the activity limitations of Morquio A patients should be regularly conducted. The Functional Dexterity Test (FDT), a validated and timed pegboard test that is easy to administer, assesses the ability to perform functional daily tasks that require a three-jaw chuck prehension pattern (also referred to as the palmar pinch, pencil pinch, or tripod grip), such as writing and buttoning [33, 34]. Patient self-report questionnaires designed to evaluate the impact of hand function on health-related quality of life are also recommended to guide treatment strategies. One such tool that is widely used for multiple hand and wrist conditions is the Disabilities of the Arm, Shoulder and Hand (DASH) questionnaire [35, 36]. Disease-specific outcome tools are likely to yield more useful information. A questionnaire has recently been developed and used by hand surgeons and therapists to evaluate limitations in activities of daily living in Morquio A patients [12].

## Recommended assessments

Once diagnosed, Morquio A patients should undergo regular assessments to evaluate the severity and progression of lower and upper extremity involvement. Recommended assessments are presented in Table 1. Ideally, the frequency of clinic visits and assessments should be tailored to meet the individual needs of each patient.

**Table 1:** Recommended assessments for evaluating and monitoring lower and upper extremity involvement in Morquio A syndrome

Assessment	At diagnosis	Annually	As clinically indicated
Lower extremities			
Physical examination	X	X	
AP pelvis radiograph^a^	X	X	X
AP standing radiographs	X		X
2-D computed tomography (CT) for rotational alignment			X
2-D CT for pre-operative planning			X
MRI			X
Arthrography			X
Formal gait analysis			X
Upper extremities			
Hand and wrist radiographs	X		
Joint range of motion	X	X	
Grip strength	X	X	
Pinch strength	X	X	
Functional assessments	X	X	

^a^AP pelvis radiographs should be obtained annually until skeletal maturity, and then as clinically indicated

## Interventions

### Lower extremity

Lower extremity alignment and hip joint correction are among the most common interventions for this patient population [7]. It is generally assumed that many patients with Morquio A syndrome will develop arthrosis requiring total joint arthroplasty of the knees or hips. However, no studies of the true natural history of this disease currently exist. Consequently, treatment is directed at the maintenance of lower extremity alignment and joint congruity. Care should be taken to not surgically alter the anatomy in these patients in such a way that will complicate the ultimate need for hip or knee replacement.

#### Hips

Hip reconstruction can be helpful in treating and postponing hip pain. The reconstruction may include either acetabular osteotomy or femoral osteotomy for less severe deformities [37], but a combination of acetabular and femoral osteotomies is typically required to correct hip subluxation in patients with classical disease [10]. Hip reconstruction can be challenging in Morquio A patients due to a shallow acetabulum. Augmentation of acetabular bone stock and customized implants are often required. As hip replacement appears to be inevitable in these patients, the goals of pediatric hip reconstruction should include improving the longevity of the native hip while respecting the anatomy of the hip in order to reduce the challenges of future hip replacement procedures. As some early experience with the Pemberton, Salter or femoral varus derotation osteotomies alone resulted in reoperation for failure to contain the femoral head, a recent report [10] advocates the use of large shelf arthroplasties which have resulted in better success in maintaining containment than Salter or Pemberton osteotomies [10, 38]. Cortical grafts from the inner table of the ilium were used, providing large grafts with a concave cortical surface to ensure improvement of the overall coverage of the acetabulum. Concomitant femoral varus de-rotational osteotomies may be needed to improve the neck shaft angle closer to 125º in these patients. In individuals who are borderline ambulant prior to surgery, it may be difficult to regain their previous level of mobility after such surgery. It is critical not to overcorrect with varus, as these patients tend to develop a waddling gait due to abductor shortening. Overcorrection with varus also functionally increases hip adduction, thus emphasizing their genu valgum, which compromises their walking ability further [10, 28]. Total hip arthroplasty may be an option for young adult patients with pain-limiting hip disease that cannot be repaired using traditional reconstructive techniques [39–42].

#### Knees

Guided growth appears to be a reliable and effective technique for correcting genu valgum in young patients with adequate growth potential (Fig. 2). The principle of guided growth relies upon temporary growth modulation of the physis using a tension band plate [43]. Typically, both the proximal tibial and distal femoral physes are addressed; however, if the deformity is confined to only one of these sites, then the procedure is performed in isolation as indicated. While this surgery may be performed as an outpatient procedure, an overnight stay is recommended for patients with this complex disorder.

Because growth is retarded in patients with Morquio A syndrome, correction takes longer than typically expected, particularly if the deformity is significant. Leaving the plates in situ for 2–3 years may be necessary, but does not appear to be detrimental. There have been no reported cases of permanent growth arrest associated with this procedure. Treatment should be initiated as soon as the deformity is observed, which may necessitate surgery in patients as young as 4 years of age. Repeated interventions should be anticipated as these children grow. It is currently unknown whether this technique has the ability to correct the most severe deformities. Although there may be concerns regarding whether the hypoplastic bone in these patients will support the screws inserted during plate hemiepiphysiodesis, this does not appear to be a problem.

In situations of severe deformity or limited remaining growth potential, growth modulation techniques may not be effective. In these circumstances, the only surgical solution is an osteotomy. Osteotomy may be performed acutely and the knee subsequently stabilized with plates and screws. Alternatively, gradual correction using a uniplanar or multiplanar external fixation frame may be employed. Both acute and gradual corrections are associated with potential complications. Acute correction risks injury to the common peroneal nerve. Bones are generally small and osteopenic [44], and subsequent fixation with implants (plates and screws) may be difficult. Recovery from acute operations can be prolonged, and in patients at risk of losing the ability to walk prior to surgery, it is possible that they may not return to their preoperative functional level.

One of the implications of improved management of the knee deformity is that patients will continue to walk and their hips and knees may then become increasingly symptomatic. If so, consideration for joint arthroplasty may be necessary. Total knee arthroplasty should be considered for patients with advanced arthrosis [45, 46]. Very few reports exist in the literature regarding arthroplasty in severely involved Morquio A patients. Custom made implants should be anticipated.

#### Ankle and foot

Ankle valgus and foot deformities can generally be managed by orthotics. Surgical options to correct ankle valgus include guided growth for smaller deformities in young children with adequate growth potential (via screw hemiepiphysiodesis) and osteotomy for larger deformities in older patients [10]. However, ankle valgus may recur. Surgical correction of the foot deformity is seldom required, but orthotics may help in a symptomatic equinovalgus foot.

### Upper extremity

Treatment for upper extremity manifestations should be guided by the functional status of the patient. Non-operative interventions include external splints to stabilize the wrist and to add strength, but children typically do not tolerate wearing them as they are usually bulky and inhibit function. The current surgical options for treating upper limb manifestations of Morquio A syndrome include permanent stabilization (for example, wrist fusion or partial fusion). Complete wrist fusion may not be optimal, as these patients rely on some residual wrist movement that is essential for key functions such as bed to wheelchair transfer. Soft tissue stabilization or partial fusions might present better surgical options in the future for treating these patients. Functional assessments should be performed on a regular basis to monitor changes in activity limitations and upper limb functioning over time.

## Peri-operative management and anesthetic considerations

Morquio A patients are at high risk of anesthesia-related morbidity and mortality due to cervical instability and myelopathy, compromised respiratory function and cardiac abnormalities [11, 47, 48]. Thorough pre-operative ear, nose and throat (ENT), pulmonary, cardiac, and radiological (cervical spine) assessments should be performed prior to elective surgery. Morquio A patients should be managed by experienced anesthesiologists familiar with MPS disorders. Correct positioning of the patient is critical and flexion and extension movements of the neck should be minimized. The use of continuous electrophysiological monitoring (somatosensory and motor evoked potentials) is recommended due to the cervical instability and high risk of cord compression [11, 47, 48]. Intubation and ventilation difficulties as well as the need for emergency tracheostomy should be anticipated, and personnel experienced in managing complex airways and difficult airway equipment should be readily available [11, 47, 48]. Whenever possible, multiple surgeries should be combined to minimize anesthetic frequency.

## Future perspectives

Currently, it is unknown if the interventions described herein will result in long-term improvement of the functional status of patients, or if the need for total joint replacement is inevitable. More studies to assess the long-term impact of surgical intervention will be required, underscoring the need to develop validated Morquio A syndrome-specific patient-reported outcome tools that will allow surgeons to quantify preoperative and postoperative functional status and to guide treatment strategies. Research is currently underway to develop validated outcome tools to assess upper and lower extremity functioning in Morquio A patients [12, 49]. Continuing advances in surgical techniques and devices will likely result in improved outcomes.

ERT for Morquio A syndrome will create new challenges for the orthopedic management of these patients. With potentially prolonged life expectancy, patients will likely require more frequent and more extensive orthopedic intervention. Enhanced endurance and mobility may encourage patients to adopt a more active lifestyle, and changes in patient expectations will likely impact orthopedic treatment strategies. Patient quality of life will be expected to assume a greater role in outcome studies.

The impact of ERT on Morquio A syndrome growth plate pathology remains to be seen. It has been demonstrated that the therapeutic enzyme penetrates poorly vascularized tissues, including growth plate cartilage in vivo [21]. Elucidation of the mechanisms that cause Morquio A syndrome pathophysiology may provide insight into optimal modes of therapy. Gene therapy may represent a promising therapeutic option for the future [50].

## Conclusions

The goals of orthopedic management of Morquio A patients are to minimize disability and to optimize functional status. Maintaining normal alignment of the lower limbs is crucial for preserving ambulatory ability. Achieving optimal hand function is important for performing activities of daily living. Standardized patient-reported outcome measures should be developed to assess the impact of orthopedic interventions and to enhance patient care. The availability of ERT underscores the importance of early diagnosis and timely intervention.
